# Metaphysics and Mental Disease
**A Text-book of Insanity and Other Mental Diseases* By Charles Arthur Mercier, M.D., F.R.C.P., F.R.C.S. (London : George Allen and Unwin, Ltd. Price 7s. 6d. net.)


**Published:** 1914-11-21

**Authors:** 


### Metaphysics and Mental Disease.*

In this second edition of his text-book of insanity, Dr. Mercier has addressed himself to a wider audience than he did in the first edition, which was intended primarily for students. Considerable additions have therefore been made for the benefit of these others who "devote themselves temporarily or permanently to the special study of insanity"; and Dr. Mercier correctly states that the student who is "reading merely for the purpose of preparing for a qualifying examination will be well advised to omit" a good deal of what he has included herein. The advice is good, unless the student can allow himself to be catholic in his tastes, for then he should make a point of reading carefully through even the section dealing with the "constitution of mind." Though there is much that is debatable in this text-book, anyone who will give serious study to its contents, and will conjoin with this careful observation of cases, is certain to be well equipped in regard to his knowledge of insanity. Dr. Mercier might perhaps have laid more stress upon the need for observation of actual cases: the study of text-books is of very great value as an adjunct to such observation, but it cannot take its place. He is rather too impatient of methods which do not appeal to him, and, being enamoured of metaphysical views with a consequent love for the method of introspection (p. 48), the author would rather "interpolate a treatise on logic and a treatise on epistemology" than give his sanction to the budding alienist who should desire "to fiddle with the ergograph" (p. 80). The first chapter gives an exceedingly clear and interesting account of the causation of insanity and of the various stresses which act inimically upon the nervous system. It is incidentally to be noted, however, that no mention is made of the condition of stress dependent upon war, and the resulting evil effects. On the other hand, the author deals effectively with the misconception that masturbation is "the most potent cause and the invariable accompaniment of insanity" (p. 17). This chapter and its immediate successor, which is devoted to "Conduct," are especially worthy of study, particularly the latter, because observation of disorder of conduct is of prime importance when the question of the person's insanity is under consideration. It would, however, take too long to deal seriatim with the various parts of the subject as set forth in this book. As far as the student is concerned, the two chapters mentioned and the portion which is taken up by a description of the forms, types, and kinds of insanity, and the "Legal Relations of Insanity," will well repay numerous readings. The passage in which the attributes of the "sane lunatic" are described (pp. 90-92) should not, however, be overlooked: for this type is one of the most troublesome to his relatives, and he presents a difficult problem to the practitioner. In regard to treatment, the advice given is for the most part sound and helpful. Stress is rightly laid upon the need for institutional care in cases of acute insanity (pp. 203 and 320); and on the necessity of the administration of an adequate amount of nourishment, even if the tube has to be resorted to: though we do not think that he is justified in condemning the nasal tube as a source of "many cases of gangrene of the lung." If these cases have resulted from its use—and we doubt if Dr. Mercier could justify the "many"—it rather points to the inexpertness of the operator. On the other hand, our experience certainly leads us to agree with him in his advocacy of the œsophageal tube as the more satisfactory method. Dr. Mercier's text-book achieved a wide popularity in the form in which it made its first appearance: and we believe that it will now reach that wider public for whom the author has prepared this larger volume.

* *A Text-book of Insanity and Other Mental Diseases.* By Charles Arthur Mercier, M.D., F.R.C.P., F.R.C.S. (London: George Allen and Unwin, Ltd. Price 7s. 6d. net.)

